# Economic Evaluation for Health Investments En Route to Universal Health Coverage: Cost-Benefit Analysis or Cost-Effectiveness Analysis?

**DOI:** 10.1016/j.jval.2018.06.005

**Published:** 2019-01

**Authors:** Anthony J. Culyer, Kalipso Chalkidou

**Affiliations:** 1 Department of Economics and Related Studies and Centre for Health Economics, University of York, York, UK; 2 Center for Global Development and Imperial College London, London, UK

**Keywords:** cost-benefit analysis, cost-effectiveness analysis, universal health coverage

## Abstract

**Background:**

It is an unresolved issue as to whether cost-benefit analysis (CBA) or cost-effectiveness analysis (CEA) is the preferable analytical toolkit for use in health technology assessment (HTA). The distinction between the two and an expressed preference for CEA go back at least to 1980 in the USA and, most recently, a Harvard-based group has been reappraising the case for CBA.

**Objectives:**

This article seeks to answer the question: would the use of cost-benefit analysis rather than the more usual cost-effectiveness analysis be an improvement, specifically in appraising health and health-related investments in low and middle-income countries (LMICs) as they transition to Universal Health Coverage?.

**Methods/Results:**

A selective literature review charts the welfare economics (welfarism and extra-welfarism) roots of both approaches. The principal distinguishing feature of the two is the monetary valuation of health outcomes under CBA compared with the use of health constructs such as the Quality-Adjusted Life-Year (QALY) or Disability-Adjusted Life-Year (DALY) under CEA. The former enables direct comparison of the outcomes of health investments with the monetized outcomes of other investments, while the CEA approach facilitates direct comparisons with other health investments. Seven challenges in using CBA in developing countries arise, including ethical issues in outcome valuation, practical challenges in the acquisition of data, intrinsic bias in data on values, and some of the practical issues of implementation for either CBA or CEA.

**Conclusions:**

We conclude with a list of nine issues that both CBA and CEA need to settle if they are to be useful in LMICs. For the immediate future we judge CBA to be the less practicable.

## The Problem

The issue with which this article concerns may be put quite simply: Would the use of cost-benefit analysis (CBA) rather than cost-effectiveness analysis (CEA) be an improvement in appraising health and health-related investments in low- and middle-income countries (LMICs) in the context of their journey toward universal health coverage (UHC)? If the answer is “yes,” then it would seem desirable to seek an international consensus on the appropriate methods to be used through the development of a best practice guide as well as a set of criteria for deciding when CBA would serve policymakers’ needs better than CEA. Here, we discuss the advantages and weaknesses of the CBA approach and propose that for policymakers interested in successfully attaining and sustaining UHC, CEA may be a more appropriate approach to economic analysis to the extent it is better suited to address the UHC objectives of maximizing health and financial protection. We conclude that for a guide to best practice for CBA to be useful, more empirical evidence is needed of whether and how policymakers use CBA to inform resource allocation decisions, followed by a process that proactively involves LMIC budget holders in its development.

We start with a review of best practice guides in the field of health economics.

## Good Practice Guides

Guides to best practice for CEA can be traced back at least as far as 1980, with the publication of “The Implications of Cost-Effectiveness Analysis of Medical Technology” [1], with subsequent editions in the initiative of Senate Committee on Labor and Human Resources and commissioned by the then Office of Technology Assessment. Other significant landmarks were made in 1994 by the Canadian Coordinating Office for Health Technology Assessment [2]; in 1987 by Drummond et al. [3] (with revised editions in 1997, 2005, and 2015); in 1996 by Gold [4] (the study that introduced the term “reference case” in health economics); in 2003 by the World Health Organization [5]; in 2004 by the National Institute for Clinical Excellence [6]; and in 2016 by the International Decision Support Initiative (iDSI) Reference Case [7], which has further details on its Web page [8] on the Reference Case development process, and complementary research. All these guides recommended CEA as the method of economic evaluation when allocating scarce health care resources.

The iDSI Reference Case for economic evaluation was funded by the Bill and Melinda Gates Foundation and was underpinned by a review of published economic evaluations in LMICs from 2000 to 2013, covering the foundation’s four focus program areas: vaccines, tuberculosis, HIV/AIDS, and malaria [9]. The review also identified the foundation as the largest single funder of economic analyses for LMICs in these areas, having commissioned almost a quarter of studies during the 13-year period studied. Only 2 of the 204 studies were described as CBAs, a finding that justified the focus on CEAs. There was substantial variation in the methodologies of the CEAs. This variation strengthened the case for developing a standardized reference case for economic evaluation, reflecting both the foundation’s and the LMIC policymakers’ needs while adhering to best methodological practice when such consensus had been or could be reached. The reference case itself was not, of course, meant to be a reference case for CBA although its broad principles as well as the process of its development could be of relevance to CBA.

Whether one needs to go further than the iDSI Reference Case when thinking about economics in LMICs is therefore a timely question. A workshop relating to another project, also funded by the Bill and Melinda Gates Foundation, was convened in 2017 to initiate a process for developing a reference case for CBA. This work began with a scoping report on the desirability of supplementing (or replacing) CEA methods with CBA, with particular reference to economic evaluations of healthcare interventions in LMICs [10].

To consider the merits of this effort requires a clear understanding of what it is that differentiates CEA and CBA and whether either has limitations, real or perceived, inherent as matters of convention, theoretical as matters of principle, or practical as matters of feasible applicability, that make one a less useful instrument than the other for appraising health care technologies in LMICs. This is what we set out to do herein.

The traditional assumptions of welfare economics that underlie the principles of conventional CBA have always proved to be something of a straitjacket when trying to apply them to real-world problems, and nowhere more than in health economics. At the root of many of the market imperfections in health care lies the ethical quandary of the weight to place on individuals’ values (which may be irrational in the sense of inconsistency with the axioms of expected utility theory, or ill-informed, or lost in a confusion of principal-agent relationships) and the selection of a means of expressing them. This arises specifically in the valuation of “health,” which is not directly traded in markets or other forms of social interaction and requires a lot of thought about the meaning and the measurement of health, its valuation and the balance to be struck between individual expressions of the meaning and its value, and collective ones. Arising at least in part from these challenges, nontraditional approaches to CEA and CBA have been developed, such as practical CBA [11], capabilities [12], new foundations [13], and extrawelfarism [14]. These have developed, although rarely in parallel, with the emergence of a host of variants, especially of CEA: behavioral cost analysis, budget-impact analysis, comparative effectiveness research, cost-consequences analysis, cost-efficiency analysis, cost-minimization analysis, cost-per-quality-adjusted life-year analysis, cost-value analysis, distributional CEA, extended CEA, generalized CEA, health technology assessment, intervention CEA, relative effectiveness assessment, sectoral CEA, and health intervention and technology assessment. Each of these has its motivation a desire to escape from (some of) the limitations of traditional welfare economics.

We applaud attempts to release economic evaluation from the straitjacket of welfarism and to devise forms of analysis that are practical, that have acceptable ethical foundations, that enable analysts to help decision makers to determine rational and consistent priorities in health care, and that address the issues they confront and embody the values that they cherish. We begin with a short review of how best practice in economic evaluation has been conceived and then, with the context of LMICs chiefly in mind, explore the differences between the two broad categories of CEA and CBA and their implications for analysts seeking best practice.

## Differences of Principle between CEA and CBA

The (near) equivalence of CEA and CBA as applications of standard welfare economics has been well documented [15], [16], [17], [18]. It is not our intention to add to this literature. The differences with which we are concerned are twofold: 1) pragmatic differences that relate to the fundamental sources of *value* for collective decision making (as when designing clinical guidelines or benefit packages in a health insurance scheme) and the methods through which values may be best revealed and 2) pragmatic differences in the *scope* of analyses (as when considering whether to monetize costs and benefits or to compare the marginal productivity of public investments across several governmental spending ministries). We term these differences “pragmatic” because they do not emanate from inherent economic theoretical underpinnings but rather from the context of application. Both CEA and CBA may be built on standard assumptions about individual welfare (utility) functions, and either may be truncated on grounds of the cost of obtaining information. As a matter of practice, advocates of CBA in health care are inclined to obtain values as directly as possible from the users of health care, whereas advocates of CEA tend to use indirect values, for example, those of publicly accountable (to users of health care) decision makers, with representative consumer values (specifically those of the general public, including patients and potential patients) mostly embodied in outcome measures such as quality-adjusted life-year (QALY). The practical, as well as ethical, question underlying this difference in approach to values concerns which one best safeguards the users’ interests, to which the answer could be either CEA or CBA. The practical question underlying the difference in approach to scope concerns which one is most fit for the specific purposes of a particular study—the study question and the context in which it is to be answered. Both these practical questions arise in the context of using methods that have credibility for the public that is being served and for the political and organizational agencies in which the collective decisions are vested.

The value difference is reflected in an early case for CEA. The Office of Technology Assessment [1] defined it thus in 1980:

The principal distinctions between CEA and CBA lie in the valuation of the desirable consequences of a decision, in the implications of the different methods of that valuation, and usually in the scope of the analysis. In CBA all costs and all benefits are valued in monetary terms. Thus, conceptually, CBA can be used to evaluate the “worth” of a project and would allow comparison of projects of different types (such as dams and hospitals). In CEA, the health-related effects of programs or technologies are not valued in monetary terms but rather are measured in some other unit (such as years of life gained). A CEA, therefore, does not result in a net monetary value for a project. Instead, it produces a measure of the cost involved in attaining some desirable health-related effect. Conceptually CEA permits direct comparison of only those programs or technologies that share similar objectives.

Comparison between programs sharing similar objectives is plainly facilitated if they have common outcome and input measures. Inputs are commonly measured in monetary units in both CEA and CBA, whereas outcomes in CEA are usually measured with a generic measure of impact on health or disability, such as life-years, QALYs, or disability-adjusted life-years (DALYs). These generic measures are plainly value-laden and a large literature explores the measures (and some work goes so far as to assert under stringent conditions that these outcome measures may be interpreted as utilities, which has given rise to a class of CEA commonly called cost-utility analysis) [19].

The fact that CEA does not value outcomes in monetary terms does not mean that comparisons cannot be made with non–health-related investments, only that they cannot be “direct” in terms of the dollar value placed on net outcomes. For example, the Second Panel on CEA [20] following on from Gold [4], and perhaps in an attempt to bridge the two approaches, recommends an “impact inventory table” to identify non–health-related effects of interventions considered in a CEA, in sectors such as education, criminal justice, the environment, and general productivity [21]. The entire edifice of economics is built upon the proposition that people can indeed make comparisons and rank preferred bundles of heterogeneous entities. *Credible* monetized indicators of value plainly facilitate comparisons between health-related intervention technologies and non–health-related interventions such as transport, education, or defense. But they have to be credible. If they are, this may constitute a positive argument for using CBA. Nevertheless, such monetary value comparisons require not only that the health investments be valued credibly in monetary terms, but also that the comparator outcomes, analogous to QALYs or DALYs, generated in other sectors and by other ministries be valued in monetary terms. So, any advantage that CBA has over CEA in terms of comparability is also enormously demanding in the information it requires elsewhere in the economy. Moreover, we argue [22] that such intersectoral comparisons may undermine ring-fenced health care budgets in emerging economies or countries transitioning out of aid dependence compounded by the fact that foreign aid has traditionally favored health care (as opposed to education or agriculture).

Is CBA in principle more comprehensive than CEA? Both CEA and CBA enable direct comparisons between investments with common measures of outcome. These need not be unique. Health investments not only generate improvements in health but can have other measurable effects as well. DALYs or QALYs may serve for the former, but the latter may come in various forms not readily measured in DALYs or QALYs, such as impact on inequalities, financial protection, or improved general productivity. There may also be health-related and non–-health-related impacts on persons who are not the direct recipients of health care, such as family members or, in the presence of externalities, members of the general public through reductions in risk from communicable diseases or empathetic satisfaction at benefits received by others. Some of these other benefits may be measurable in principle by DALYs or QALYs (e.g., impact on family members, herd immunity) but others will need other metrics, some monetary (e.g., productivity effects) and some nonmonetary (e.g., more time in school). And while the net production impact can be added on to the value of the net QALY gains from an intervention [23], the more the benefits added, the more CEA may come to resemble some versions of multicriteria decision analysis [24], [25].

A need for a diversity of outcome measures is a natural consequence of any intervention that has multiple impacts, whether or not they are to be valued in monetary terms. Although the diversity certainly adds to the complexity of a CEA, it does not require the monetization of the measures in question. It is possible to describe the outcome of interventions as a bundle of effects, whose valuation will be made easier given qualitative and quantitative measures (say, of health inequalities), but whose monetization in terms of collective willingness to pay for their delivery may add little useful information for decision makers to that already added by the measures themselves. There is therefore no reason of *principle* for excluding these effects from CEA studies, other than practicability, for example, to exclude such effects altogether unless they are deemed likely to be large or to be likely to turn an incremental cost-effectiveness ratio (ICER) to one side or the other of a critical threshold.

CEA, like CBA, is not designed to provide final answers to complex policy choices. Both offer, instead, a way of thinking about them. CEA and CBA are aids to thought, not substitutes for it. As Sugden and Williams [11] put it:

… the role of the analyst is to assist, not simply a decision-maker, but a decision-making process that has the assent of the community as a whole. The decision-maker is responsible for making a decision, according to his own lights, but he is responsible to the community. His right to decide stems from the consent of the community, expressed through the political system. The community, then, ought to have the right to call upon the decision-maker to account for his decisions.

So long as quantification of effects is viewed as helpful to decision makers, the fact of their having heterogeneous measures, some real and some monetary, cannot be a ground in principle for rejecting CEA studies in favor of CBA. A good CEA will alert decision makers to all the relevant issues and present them in an orderly fashion that facilitates judgment and choice. The questions of what is relevant, of what is “in” and what is “out,” is a matter of judgment for the *decision maker*, who may be guided by analysts as to the kinds of measures that are available and the likely cost of obtaining information that is not currently available. The methodologies of CEA and CBA do not, as a matter of principle, include or exclude. That is what a scoping exercise does for any study, and scoping decisions are for the decision maker to make.

## Sources of Value or Willingness to Pay

The fact that CBA involves the monetary valuation of health and other outcomes has no implications in and of itself about the source of such valuation. It is sometimes argued that the source of collective willingness to pay ought, as a matter of principle, be individualistic. It is argued, for example, that [10]:

Benefit-cost analysis… is based on the idea that each individual is the best (or most legitimate) judge of how a particular consequence affects his or her wellbeing, and combines effects on multiple individuals by adding their monetary values for the changes. The reliance on individual preferences respects individual autonomy. The logic of the aggregation is that increasing the population sum of net benefits increases the available set of goods and services that affect individuals’ wellbeing, and hence creates the possibility that everyone will be better off.

It is certainly possible to propose a *particular* form of CBA of which this would be true. Nevertheless, it presents the difficulty of how we would describe an approach to collective monetary valuation that was *not* based on individualistic valuations. In fact, we know that there are many good grounds for suspicion about individual strengths of preference or willingness to pay for interventions. In most countries of the world, whether LMICs or high-income countries (HICs), the dangers to welfare of the individualistic way of valuing health benefits are recognized by a host of regulatory and consultative processes designed to optimize the deployment of evidence-informed and client-friendly clinical expertise in the pursuit of population health. Health care is notorious for being a territory in which virtually none of the usual conditions for efficient markets apply: the socioeconomic gradient linking health and wealth (willingness to pay being correlated inversely with ill-health and disability and hence with most notions of need), principal-agent imperfections (as when doctors’ prescriptions and treatments accord more with their financial interests than with the patient’s need for medication or with best practice guidelines, including supplier-induced demand), asymmetrical information (as when such knowledge as the professional has is not shared with the patient, and the patient’s own expert knowledge of their personal and family circumstances is ignored), ignorant and prejudiced medical judgments taken without regard to any evidence of benefit to the patient, irrational behavior (whether by the professional or by the patient), patient incompetence to decide through youth or old age, externalities (physical and psychic), and publicness (in the technical economics sense as when individuals cannot be excluded from receipt of services such as clean water or unpolluted air) to name the most common. The bearing of each of these on the reliability of an individual’s estimate of the value to them of specific interventions, or of their expected consequences, varies. Collectively, however, they present a demanding set of hurdles to be cleared before these individual expressions, or their aggregation, may be regarded as an acceptable representation of the public interest.

An alternative source for making high-level decisions about public willingness to pay might instead be panels that were broadly representative of the communities on the behalf of which they were acting, and which were supported by expert advisers. Rather than seeking to monetize all outcomes (supposing that this were feasible and acceptable), CEA does nonetheless require the expression of one critically important value. This is the “threshold” ICER above which no intervention will be included in the package of benefits (at least not without persuasive arguments to the contrary). It is also generally accepted that critically important value components of CEA, such as the construction of QALYs or—less frequently—DALYs, ought to be informed by patients’ values.

In any event, CBA is not, as a matter of principle, wedded to the use of the classic individualistic rhetoric of neoclassical economics. Nor is it necessarily wedded to a utilitarian maximand. CEA cannot escape some monetization of outcome—the threshold (either explicit or implicit). One commonly finds that health gain and a concern for a fair distribution are the objects of policy, as with sustainable development goals or in UHC. In such cases, a form of CBA that did not mesh with those values would not be helpful. An attraction of CEA is that the threshold ICER is transparently and directly related to a common objective of all health care systems and, as an idea, is every bit as transferable across countries and health systems as money. It facilitates transfer of evidence and economizes on global analytic efforts especially as the literature regarding deriving empirical threshold in different countries is growing [26].

Table 1 presents some of the challenges of applying CBA in the context of countries transitioning toward UHC. These are in every case additional to any conceptual or empirical challenges posed by CEA.

**Table 1:** Seven challenges in applying CBA to inform countries’ investments en route to UHC

1. A fundamental conflict of value between outcomes in CEA, which are valued broadly equally whoever receives them, and outcomes in CBA, which are valued according to ability to pay. 2. A further fundamental conflict of value is that UHC requires coverage regardless of individual ability to pay, and so the use of CBA may bias the selection of services to be made available in ways that are contrary to the interests of patients with lower incomes. 3. CBA entails acceptance of an implausible set of assumptions about the invariable good judgment of both patients and medical professionals. 4. The elicitation of consumer (and other stakeholder) monetary valuation of health-related outcomes is fraught with experimental, framing, and many other potential biases, all which are additional to those already inherent on QALY or DALY measurement. 5. CEA is, as a matter of principle, more transparent and transferable across countries and health systems as it offers a universal indicator for priority setting, like the health gain per dollar of expenditure, which depends neither on (a) individual abilities to pay nor (b) a presumption that ability to pay is the same in all societies. 6. CEA works particularly well when there exists a credible threshold for decisions at the margin, which in practice will be what most decisions require. 7. CBA requires valuation of the outputs of interventions in all other sectors against which the value of health care interventions can be compared. This is simply impractical for the foreseeable future.

CBA, cost-benefit analysis; CEA, cost-effectiveness analysis; DALY, disability-adjusted life years; UHC, universal health coverage; QALY, quality-adjusted life year.

## Data and Other Informational Differences between CEA and CBA

The informational requirements of CEA can be formidable but those of CBA are typically even more demanding, because they add the task of monetizing real and in-kind effects of interventions to the task of measuring their costs and quantifying their effects.

The use of both CEA and CBA in LMICs usually confronts formidable practical issues, of which those presented in Table 2 are often encountered.

**Table 2:** Nine issues for both CEA and CBA in LMICs

1. The relevant data are not local; are incomplete, unreliable, or imprecise; are challenged by experts; or are completely absent. 2. High quality analytical capacity is lacking in clinical disciplines, health economics, epidemiology, ethics, biostatistics, systematic reviewing and meta-analysis. Although all of these disciplines are essential to the professional conduct of CEA and CBA more of them are challenging for the purposes of CBA than for the relatively modest CEA. 3. Measures of outcome comparable to QALYs or DALYs are absent, along with their monetary valuations, rendering the calculation of valuations of QALYs and DALYs irrelevant. 4. Eliciting and using experimentally derived outcome valuations is a major research exercise fraught with potential biases over and above those entailed with DALY or QALY measurement and not to be cheaply replicated in every country. 5. It is not uncommon to find a prejudiced and unsympathetic, but dominant, senior cohort of professionals whose cooperation is less likely given the particular demands of CBA relative to CEA. 6. Political understanding of the value of pricing outcomes is likely to be absent. 7. Gaining acceptance of a threshold is already a tough task, extending it to valuing many outcomes, including those in nonhealth sectors, could at times be at odds with the international movement towards current SDGs and UHC. 8. Public understanding and acceptance of a solution modelled on a theoretical market outcome based on individuals’ ability to pay is unlikely. 9. Major foreign funders of health care and health care policies have their own disease, or technology, or population level priorities and budgets not based on CBA or CEA.

CBA, cost-benefit analysis; CEA, cost-effectiveness analysis; DALY, disability-adjusted life years; QALY, quality-adjusted life year; SDG, sustainable development goals; UHC, universal health coverage.

Collectively, these factors militate against sophistication and fine-grained analyses. They also run against the value system underlying UHC. What is needed are analyses that are consistent with, and readily understandable and communicable in, the context in which they are to be used.

These are all substantial challenges for CBA. Purely on pragmatic grounds, therefore, CBA may be an unmanageable further step in many LMICs, and even if it were manageable, it may offer little additional advantage over a more modest CEA. A form of CBA that denied the legitimacy of health maximization and financial protection as policy goals would certainly not be helpful, whereas one that entrenched existing inequalities in society militates against the very purpose of UHC.

## Who Is in Charge?

Nevertheless, it would be wrong to *presume* that CBA is a step too far. Because the choice of CEA or CBA is at root a choice of the scope of a study, it is again a matter for decision makers. Their choice will depend on their chosen maximand and their social value judgments. It will also depend on the nature of the choice confronting them. A choice, for example, that may favor the use of CBA, involving investment in health care or another sector, such as education or housing, is one in which a common dollar unit of valuation may be helpful. Nevertheless, as noted earlier, this would entail valuing not only health outcomes in terms of dollars but also the outcomes of the comparator sectors (i.e., education or housing). Most LMICs (indeed, most HICs) are a long way short of having an ability to make such valuations, at least for the foreseeable future. Although in some HICs such valuations have existed for some time in the fields of transport and environmental policy, they scarcely exist at all in LMICs. In the social services in particular, outcome measurement is still relatively primitive and heterogeneous in all countries, production functions are disputed, and assigning monetary values of international relevance would assuredly be riven with controversy. For the foreseeable future, therefore, CEA seems to us the practical route to take. The time for CBA may come but much basic research spadework remains to be done. Perhaps the guide to best practice for CBA can help by highlighting an empirical research agenda both to better understand policymakers’ needs and to help address these.
